# The GENIE Data Model: A Solid Tumor, Person-Centric Framework for Scalable, Harmonized Precision Oncology Data Collection

**DOI:** 10.1158/2767-9764.CRC-26-0148

**Published:** 2026-09-23

**Authors:** Jennifer N. Hoppe, Jocelyn Lee, Tomi F. Akinyemiju, Danielle S. Bitterman, Michael Brudno, Michelle F. Green, Vojtech Huser, Jafi A. Lipson, Sanjay Mishra, Daniel P. Nussbaum, Jai N. Patel, Valentina I. Petkov, Kelli M. Rasmussen, Sahussapont Joseph Sirintrapun, Patricia A. Spears, Sebastiaan Van Sandijk, Anne-Marie Meyer, Umit Topaloglu, Jeremy L. Warner

**Affiliations:** 1 American Association for Cancer Research: Project GENIE, Philadelphia, Pennsylvania.; 2 Bristol-Myers Squibb, Princeton, New Jersey.; 3 Duke Cancer Institute, Duke University, Durham, North Carolina.; 4 Department of Population Health Sciences, Duke University Medical Center, Durham, North Carolina.; 5 AstraZeneca, Gaithersburg, Maryland.; 6 Genetics and Genome Biology, SickKids Research Institute, Toronto, Canada.; 7 Department of Computer Science, University of Toronto, Toronto, Canada.; 8 Labcorp, Burlington, North Carolina.; 9 EPAM, Cambridge, Massachusetts.; 10 Stanford University School of Medicine, Stanford, California.; 11 Center for Clinical Cancer Informatics and Data Science, Legorreta Cancer Center, Brown University, Providence, Rhode Island.; 12 Brown University Health Cancer Institute, Rhode Island Hospital, Providence, Rhode Island.; 13 Department of Surgery, Duke University, Durham, North Carolina.; 14 Division of Cancer Pharmacology and Pharmacogenomics, Atrium Health Levine Cancer, Charlotte, North Carolina.; 15 Atrium Health Wake Forest Baptist Comprehensive Cancer Center, Winston-Salem, North Carolina.; 16 Wake Forest University School of Medicine, Charlotte, North Carolina.; 17 Surveillance Research Program, Division of Cancer Control and Population Sciences, National Cancer Institute, Bethesda, Maryland.; 18 Mass General Brigham, Boston, Massachusetts.; 19 The University of North Carolina at Chapel Hill, Chapel Hill, North Carolina.; 20 Patient Advocates for Research Council, UNC Lineberger Comprehensive Cancer Center, Chapel Hill, North Carolina.; 21 OHDSI Collaborators, Observational Health Data Sciences and Informatics (OHDSI), New York, New York.; 22 Center for Biomedical Informatics and Information Technology, National Cancer Institute, Rockville, Maryland.

## Abstract

Adequately powered analyses in precision oncology often require combining cohorts across institutions. However, integration is constrained by the least granular source and may become infeasible when data elements are too heterogeneous to harmonize and map to a common data model. This challenge is acute in multi-institutional precision oncology research, in which real-world evidence requires harmonized clinico-omic data integration. Existing models often lack sufficient treatment patterns, outcomes, and genomic data, limiting interoperability and scalability. To address these gaps, American Association for Cancer Research Project Genomics Evidence Neoplasia Information Exchange (GENIE) developed the GENIE Data Model (GDM), a comprehensive, open-source oncology data model for scalable, consistent, and interoperable data collection across solid tumors, designed to effectively capture the journey of patients with cancer. Through iterative consensus-building, four working groups comprising 13 subject matter experts defined data elements across multiple clinical domains: patient characteristics, imaging, diagnosis, surgery, histopathology, biomarkers, systemic therapy, radiation, clinical trial history, disease response and outcomes, and social determinants of health. Elements were defined using standardized terminologies and permissible values to support mapping to Health Level 7 Fast Healthcare Interoperability Resources, Observational Medical Outcomes Partnership, and other existing oncology standards. The model architecture distinguishes manually abstracted elements from computationally collected elements, enabling parallel workflows. The GDM provides an extensible framework that addresses critical gaps and enables scalable, harmonized data collection essential for precision oncology and real-world evidence generation.

**Significance:**

The GDM is an open-source, ready-to-implement, solid tumor framework that standardizes clinical and genomic data capture across institutions to effectively capture the journey of patients with cancer. It enables interoperable mapping to existing standards, accelerates multisite study start-up, and supports scalable real-world evidence generation in precision oncology.

## Introduction

**Figure 1.**
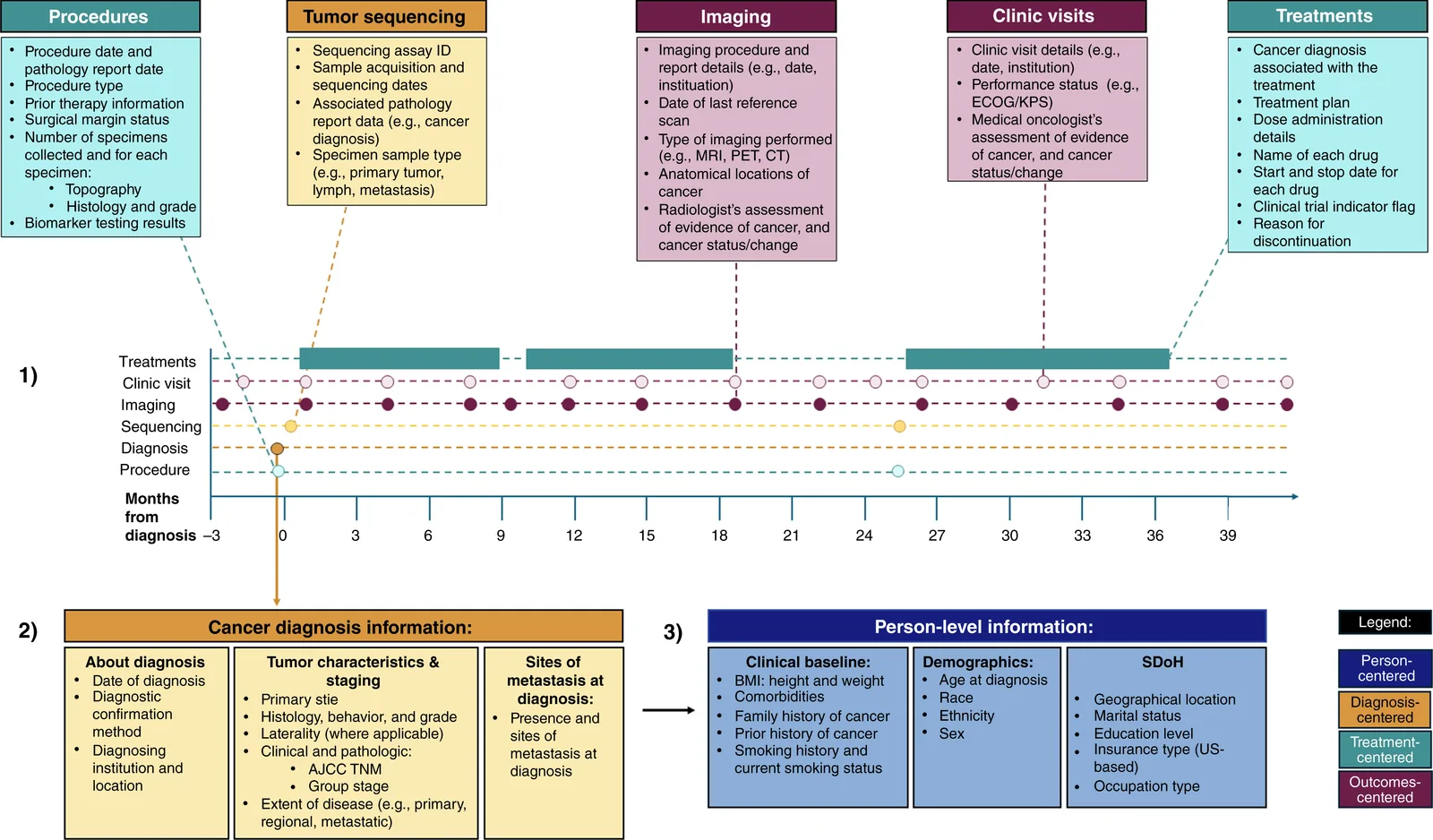
Journey of patients living with cancer. The journey of each person living with cancer is complex and consists of episodic interactions with the healthcare system, each generating clinically meaningful information about disease status and care delivery. (1) A representative person-level timeline illustrating the temporal organization of events from diagnosis through follow-up. Systemic therapies are depicted as teal bars, representing treatment intervals and duration. Discrete clinical events are shown as time-stamped points, including clinic visits (burgundy dots), imaging assessments (pink dots), tumor sequencing (yellow dots), and procedures (blue dots). The horizontal alignment of these events supports time-aware analyses of disease evolution, treatment sequencing, and outcomes. (2) Cancer diagnosis information establishes the disease-centered clinical baseline and includes the date of diagnosis, diagnostic confirmation method, diagnosing institution, primary tumor site, histology, tumor grade, clinical and pathologic staging, and sites of metastasis at diagnosis, if present. These elements anchor disease classification, risk stratification, and prognostic assessment. (3) Person-level information captures patient-centered context, including clinical baseline characteristics (e.g., body mass index, comorbidities, family and prior cancer history, smoking history), demographics (age at diagnosis, race, ethnicity, sex), and SDoH (e.g., geographic location, marital status, education, insurance type, occupation). These factors independently influence treatment access, tolerance, and outcomes. Collectively, these diagnosis-, treatment-, outcomes-, and person-centered data elements enable an integrated longitudinal view of the cancer journey that supports both direct clinical insight and secondary research use. ECOG, Eastern Cooperative Oncology Group; KPS, Karnofsky performance status; TNM, tumor–node–metastasis.

The full realization of the promise of precision medicine requires learning from every patient at each stage of their treatment journey across institutions globally (1). The journeys of patients with cancer can be complex, with numerous clinical interactions, sometimes across multiple health systems. Each clinical interaction generates copious amounts of data that require synthesis and subsequent analysis to guide patient care and clinical research, either alone or in combination with additional health system or neighborhood-level data (Fig. 1). The growth of precision oncology has resulted in large clinico-omic datasets, yet inconsistent data collection protocols limit effective integration to support real-world evidence-based research. Although substantial progress has been made in oncology data standardization, persistent challenges remain, including heterogeneous data capture practices, inconsistent semantic definitions, and gaps in existing standards. Several initiatives have emerged to advance oncology data standardization, each tailored to distinct use cases.

The Minimal Common Oncology Data Elements (mCODE) initiative, developed as a Health Level 7 (HL7) Fast Healthcare Interoperability Resources (FHIR) implementation guide, defines oncology data elements intended to improve interoperability and enable research-quality data capture at the point of care (2). mCODE has achieved moderate adoption through federal programs such as the Enhancing Oncology Model and United States Core Data for Interoperability plus Cancer (USCDI+ Cancer; refs. 2–4). Although mCODE supports patient and disease characterization, significant gaps exist in treatment patterns and health outcomes, including missing elements for treatment regimens, line of therapy, therapy discontinuation reasons, and outcome measures (5).

Utilizing FHIR-based application programming interfaces (API) and aligning with mCODE where possible, the USCDI+ Cancer extension includes oncology-specific elements for regulatory and quality reporting but is not designed to provide the granularity required for research use cases involving longitudinal treatment patterns and complex outcome ascertainment (4).

On the research analytics side, the Observational Medical Outcomes Partnership (OMOP) Common Data Model (CDM) provides a robust framework for harmonizing observational health data across heterogeneous sources, with oncology extensions introducing constructs for treatment episodes, staging, and clinical modifiers (6, 7). However, OMOP remains limited in its representation of genomic variants, complex multiagent regimens, and the evolving workflows characteristic of precision oncology practice (8). These challenges reflect a fundamental tension in oncology data standardization: Models optimized for clinical interoperability often lack the granularity required for research, whereas research-focused frameworks may not align well with clinical workflows.

**Figure 2.**
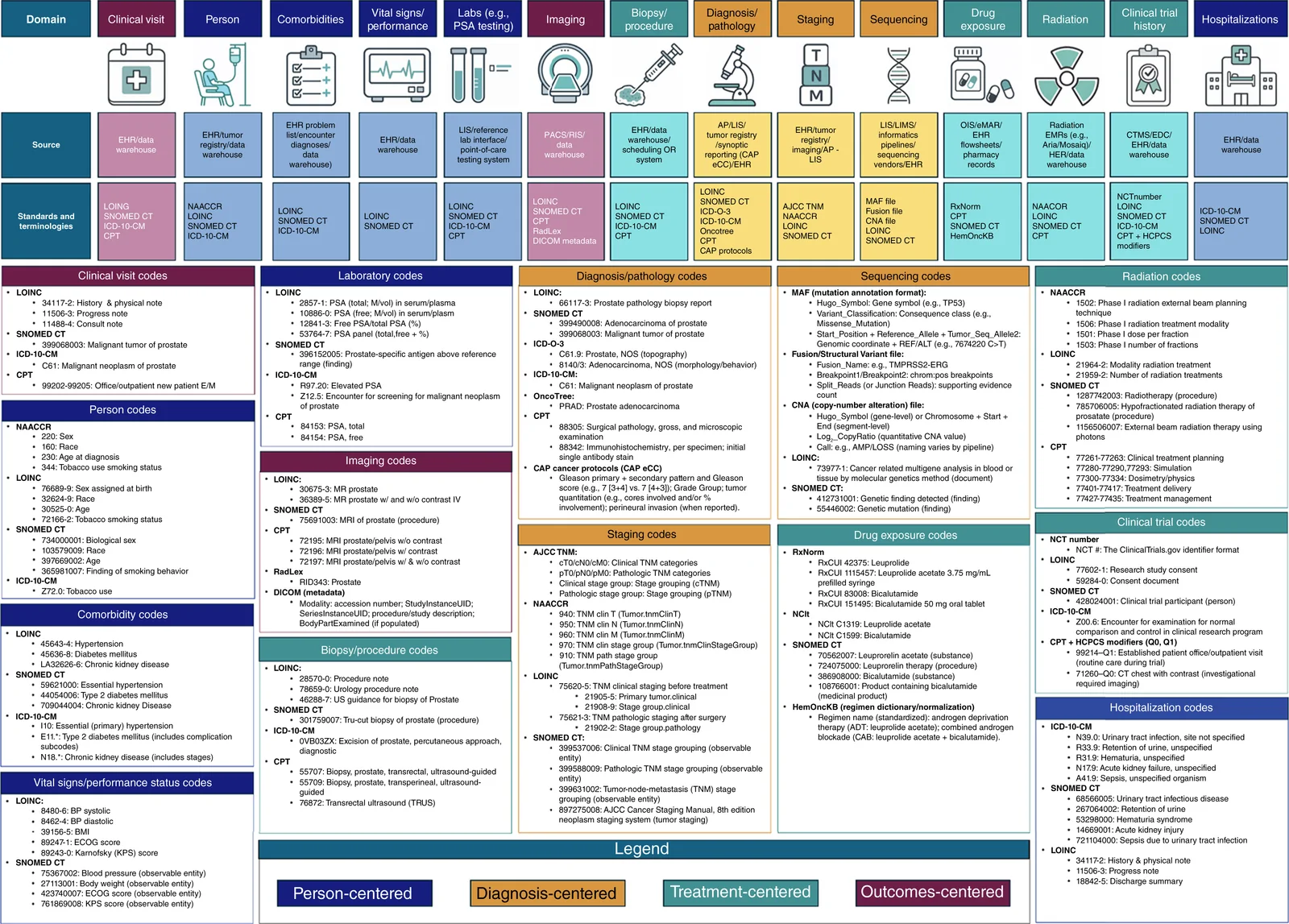
Data describing the journey of a person living with cancer. The journey of a person living with cancer is represented across multiple clinical domains, each capturing distinct aspects of diagnosis, treatment, and outcomes and each typically residing in different information systems within a healthcare organization. The figure organizes these data into three aligned layers: clinical domains, source systems, and standards and terminologies. Across the top row, representative clinical domains include clinic visits, person-level characteristics and comorbidities, vital signs and performance status, laboratory testing, imaging, biopsy and procedures, diagnosis and pathology, tumor staging, molecular sequencing, drug exposure, radiotherapy, clinical trial history, and hospitalizations. These domains span person-, diagnosis-, treatment-, and outcomes-centered aspects of the cancer journey. The middle row illustrates typical source systems from which these data originate, including EHR data warehouses, laboratory information systems (LIS), picture archiving and communication systems (PACS), pathology workflows, tumor registries, sequencing pipelines, oncology information systems, radiation oncology systems, and clinical trial management platforms. The bottom row highlights the standards and terminologies commonly used to represent data within each domain, including but not limited to American Joint Committee on Cancer, Current Procedural Terminology (CPT), HemOncKB, ICD-10-CM, ICD-O-3, LOINC, NAACCR, NCIt, National Library of Medicine database of drug terms (RxNorm), SNOMED CT; and tumor–node–metastasis (TNM) staging system. The prostate cancer example illustrates that no single standard is sufficient to fully describe disease classification, burden, molecular features, treatment intent, and outcomes. Instead, longitudinal cancer phenotyping requires integration of data generated across diverse clinical systems and standards. AP, Anatomic Pathology; CAP, College of American Pathologists; eCC, electronic Cancer Checklists; ECOG, Eastern Cooperative Oncology Group; EMR, Electronic Medical Record; HER or EHR, Electronic Health Record; KPS, Karnofsky performance status; LIMS, Laboratory Information Management System; NCT, National Clinical Trial; OR, overall Response. (Icons in this figure adapted from an image generated by ChatGPT, February 6, 2026, OpenAI, https://chat.openai.com.)

However, no single terminology provides comprehensive coverage across diagnosis, treatment, and outcomes domains. Beyond structural limitations, terminology fragmentation, in which overlapping but nonequivalent coding systems are used to represent the same clinical concepts, presents an additional barrier to interoperability (Fig. 2). Oncology-specific content spans multiple vocabularies, including the International Classification of Diseases for Oncology (ICD-O), North American Association of Central Cancer Registries (NAACCR), Systematized Nomenclature of Medicine–Clinical Terms (SNOMED CT), Logical Observation Identifiers Names and Codes (LOINC), and the National Cancer Institute Thesaurus (NCIt), as well as emerging resources such as the Hematology Oncology Knowledge Base (HemOncKB) and OncoTree (9–15). This fragmentation complicates automated data extraction, cross-project harmonization, and effective secondary use of clinical data for research. Together, gaps in data standards, defined as agreed-upon frameworks for structuring and representing clinical concepts across institutions and systems, and the lack of oncology-specific terminology consensus hinder scalable, automated data collection for real-world evidence generation, particularly in multi-institutional research initiatives such as American Association for Cancer Research (AACR) Project Genomics Evidence Neoplasia Information Exchange (GENIE; ref. 16).

To address these challenges, the GENIE Data Model (GDM) was developed as a comprehensive, open-source, solid tumor data model designed to enable scalable, interoperable clinico-omic data capture from diverse institutional systems to effectively capture the journeys of patients with cancer. GDM builds on existing standards, including HL7 FHIR, mCODE, USCDI+ Cancer, and OMOP, while introducing the extensibility and automation capabilities required for modern precision oncology research (Table 1). The GDM was developed with three primary design goals: (i) a cohort-agnostic architecture enabling consistent data capture across tumor types, (ii) support for both prospective and retrospective data collection, and (iii) extensibility to accommodate future clinical, genomic, and analytic advances.

**Table 1.** Overview of existing models and the GDM.

Feature	mCODE Standard for Trial Use 4	USCDI+ Cancer Registry v1	OMOP CDM v5.4	GENIE model v.1
Primary purpose	Interoperable exchange of core oncology data	Regulatory reporting and quality measurement for cancer	Harmonized analytics for observational research	Scalable, extensible model for research and harmonization
Underlying standard	HL7 FHIR	HL7 FHIR	Relational common data model (OHDSI)	Hybrid: aligning with FHIR and OMOP, with oncology-specific extensions
Scope	Minimal core elements to support EHR integration	Priority cancer elements supporting EHR-to-registry workflows	Broad clinical domains with limited oncology-specific extensions	Comprehensive oncology domains (patient, tumor, treatment, genomics, outcomes)
Strengths	Widely adopted; closely aligned with EHR workflows	Policy-driven standard; supporting registry reporting and secondary use	Mature analytics ecosystem; standardized vocabularies and tooling	Extensible; supporting automation; explicit cross-standard mappings
Limitations for research	Limited support for longitudinal treatment patterns, regimens, and outcomes	Limited granularity; minimal support for genomics and research use cases	Limited support for genomics and treatment regimens; requires *post hoc* inference	Limited to Solid Tumors (v.1); limited capture of patient-reported outcomes (v.1); full GDM deployment can be resource-intensive
Interoperability focus	High (FHIR-based exchange)	High (FHIR-based exchange)	Moderate (which requires ETL processes)	High (FHIR- and OMOP-aligned; supporting API-based automation)

Here, we describe the development, structure, and key features of the GDM. We detail the consensus-based process used to specify a comprehensive list of data elements across multiple clinical domains, the logical model architecture that coordinates manual abstraction with computationally collected data elements, and the alignment strategy enabling interoperability with existing oncology data standards. The complete model specification and supporting documentation are publicly available on GitHub at https://github.com/AACR-project-GENIE to enable adoption and adaptation by the broader research community.

## Materials and Methods

### Project organization and governance

The GDM was developed as a comprehensive, solid tumor, clinico-omic data model to support real-world oncology data collection and research. Although the GDM specifies clinical phenotypes, it was intentionally designed as a flexible framework for integration with multiomic data. The primary use case supports linkage to somatic next-generation sequencing data within the GENIE Consortium; however, the model also accommodates the linkage of clinical phenotypes with germline sequencing, transcriptomic profiling, and additional omics modalities, contingent upon institutional capabilities and research objectives. Thirteen subject matter experts, including medical, surgical, and radiation oncologists; radiologists; pathologists; pharmacologists; patient advocates; epidemiologists; bioinformaticians; and data scientists, were organized into four working groups: diagnosis, treatment, response/outcomes, and social determinants of health (SDoH). Working groups met biweekly and were facilitated by project co-leads A.-M. Meyer, U. Topaloglu, J.L. Warner, and AACR staff, with members participating across groups to ensure cross-domain alignment. Each working group defined relevant data elements, value sets, abstraction hierarchies, and terminology bindings. Proposed specifications underwent iterative review and were approved by consensus based on predefined criteria: (i) clinical relevance, (ii) abstraction feasibility from source documentation, and (iii) cross-institutional availability. Where benchmarking against existing data resources was not feasible, specifications were advanced through structured deliberation and agreement among working group members. A guiding principle across all working groups was to anchor the model, to the greatest extent possible, to person-centric definitions. To support this principle, patient advocates were intentionally embedded within each working group.

### Clinical domain specification

**Figure 3.**
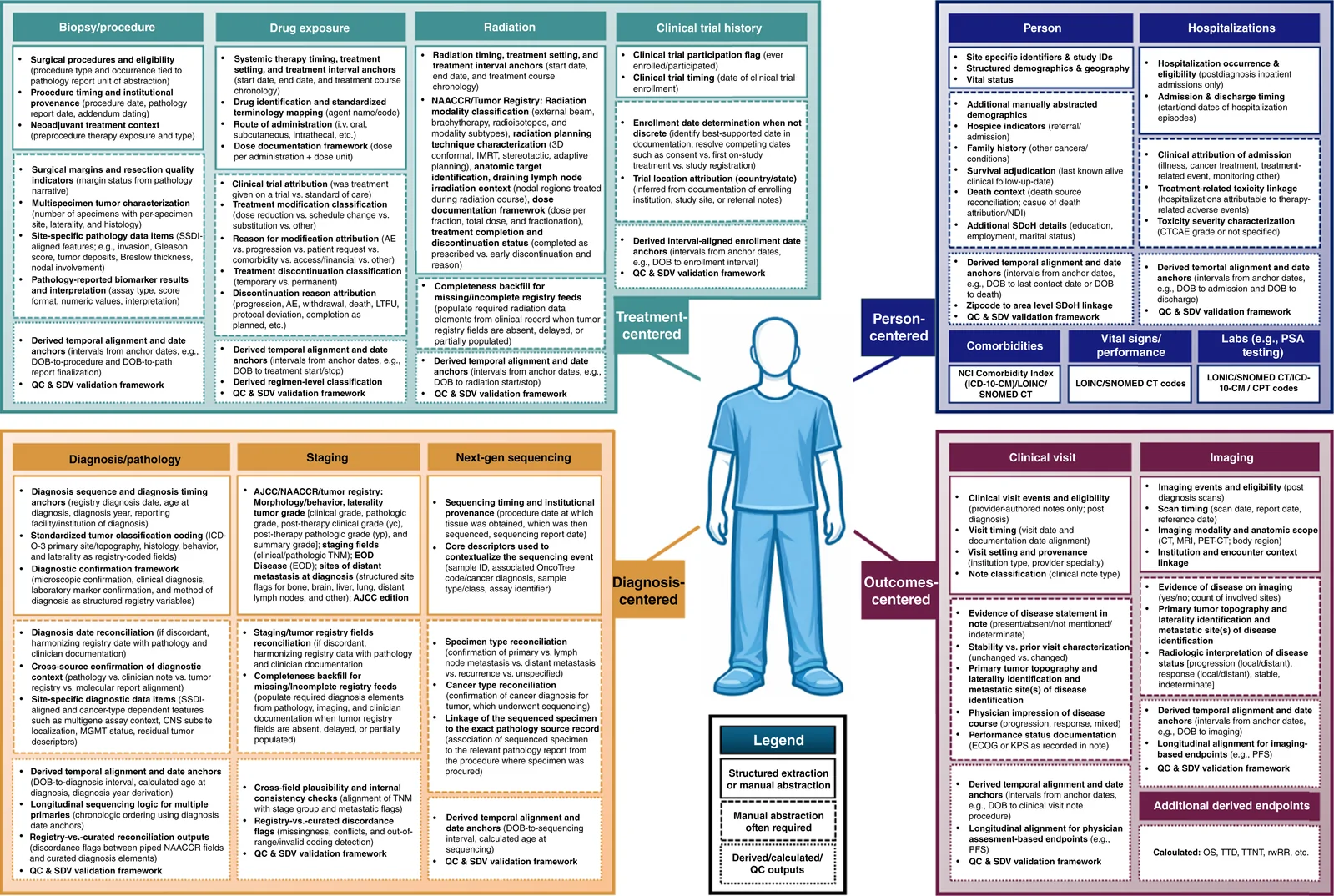
GDM domain schema. The GDM schema summarizes the full set of data elements collected to represent the journey of a person living with cancer by integrating (i) foundational structured data encoded in routine clinical systems using standard terminologies (Fig. 2), (ii) high-value variables that often require manual abstraction from unstructured documentation, and (iii) derived variables calculated from (i) and (ii). The schema organizes elements into domains centered on the patient with cancer. The person-centered domain includes demographics, geographical linkage to area SDoH, individual-level SDoH (education, employment, marital status), hospitalization events, comorbidities, vital signs/performance status, and laboratory testing. The treatment-centered domain includes surgeries (procedures and pathology reports) and radiotherapy (course/phase, technique/modality, and dosimetry), systemic therapies (drug-level exposure with start/end dates and modification/discontinuation reasons), and clinical trial participation. The diagnosis-centered domain includes cancer diagnosis (ICD-O-3 tumor classification coding; diagnostic confirmation framework), cancer staging [grade, behavior, morphology, tumor–node–metastasis (TNM)], and genomic profiling of tumors (somatic alterations and sequencing panel metadata). Lastly, the outcomes-centered domain includes imaging (event dates, imaging types, radiologist assessment of cancer status and response) and clinical visits (event dates, visit types, and oncologist assessment of cancer status and response). The outcomes-centered domain also harmonizes temporally anchored tumor-centric assessments and person-centric measures to compute real-world endpoints, including overall survival (OS), progression-free survival (PFS), time to treatment discontinuation (TTD), time to next treatment (TTNT), and real-world response rate (rwRR). Together, these domains represent the complete GDM scope and illustrate how combining coded structured data with curated abstraction enables robust longitudinal phenotyping for real-world evidence generation. AE, adverse event; CNS, central nervous system; CTCAE, Common Terminology Criteria for Adverse Events; DOB, Date of Birth; ECOG, Eastern Cooperative Oncology Group; EOD, extent of disease; KPS, Karnofsky performance status; LTFU, Lost to Follow-Up; MGMT, O^6^-methylguanine-DNA methyltransferase; NDI, National Death Index; SDV, Source Data Verification; SSDI, Site-Specific Data Item. (Icon in this figure adapted from an image generated by ChatGPT, February 6, 2026, OpenAI, https://chat.openai.com.)

**Figure 4.**
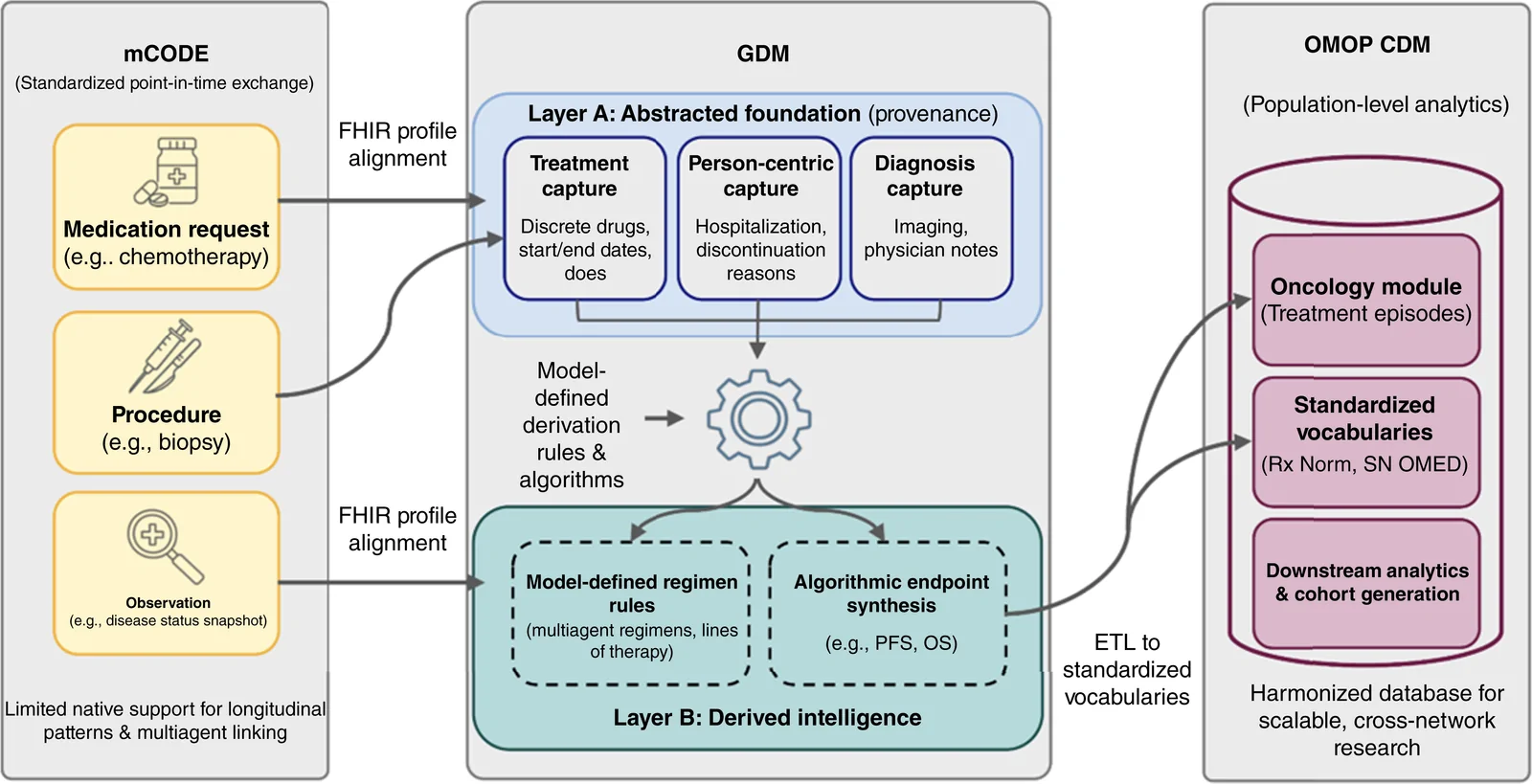
GDM interoperability. The figure illustrates how the GDM bridges standardized point-in-time oncology data exchange and population-level analytics by integrating and extending existing interoperability frameworks. (Left) The mCODE provides standardized HL7 FHIR profiles for discrete oncology concepts, including *MedicationRequest* (e.g., chemotherapy agents), *Procedure* (e.g., biopsy), and *Observation* (e.g., disease status snapshots). Although mCODE enables standardized exchange of individual clinical events, it has limited native support for longitudinal patterning and multiagent treatment constructs. (Middle) The GDM’s two-layer architecture. Layer A (abstracted foundation) captures granular, source-level clinical data aligned to mCODE-compatible FHIR profiles while preserving provenance and clinical context. This layer includes agent-level therapy exposure (discrete drugs with start and end dates and dose information), person-centric clinical events (e.g., hospitalizations and treatment discontinuation reasons), and tumor-centric assessments (e.g., imaging and clinician documentation). Building on this foundation, layer B (derived intelligence) applies model-defined derivation rules and algorithms to synthesize these granular inputs into longitudinally, analytically meaningful constructs. These include multiagent regimens, lines of therapy, and algorithmically derived endpoints such as progression-free survival and overall survival, with traceable logic linking derived outputs back to underlying source data. (Right) GDM outputs are transformed through ETL processes to support interoperability with standardized vocabularies and episode-based presentations compatible with data models like OMOP CDM. This enables population-level analytics, cohort generation, and downstream research across institutions. Together, this workflow supports the collection of heterogeneous data from a wide range of sources while maintaining transparent provenance, extensibility, and alignment with existing oncology data standards. (This figure adapted from an image generated by ChatGPT, February 6, 2026, OpenAI, https://chat.openai.com.)

Using this process, the working groups developed the GDM as a set of data elements organized across multiple clinical domains spanning the cancer care continuum (Fig. 3; Table 2). Four criteria guided element selection: (i) clinical or research importance, including alignment with National Comprehensive Cancer Network (NCCN) guidelines; (ii) likelihood of documentation in electronic health records (EHR); (iii) availability of standardized terminologies such as ICD-O, ICD, 10th Revision, Clinical Modification (ICD-10-CM), Current Procedural Terminology, Healthcare Common Procedure Coding System, SNOMED CT, and LOINC; and (iv) compatibility with tumor registry standards (9, 11, 12, 17–20). Abstraction approaches were defined for each variable, prioritizing programmatic extraction from structured EHR fields when prior literature and internal validation indicated that these sources provide a clinically faithful representation (e.g., diagnosis codes and other native, source system data elements). Manual curation was recommended for elements captured primarily in unstructured documentation, with these specifications designed to remain compatible with future automation as data capture capabilities evolve. For treatment domains, elements were designed to capture sufficient granularity to enable clear attribution of treatment modifications and discontinuations and to support downstream derivation of treatment regimen patterns. Outcome elements support both tumor-centric assessments (e.g., imaging response, physician documentation) and person-centric measures (e.g., hospitalizations, treatment discontinuations, vital status). Lastly, derived endpoints are calculated using existing rules-based algorithms that leverage disease-specific criteria and temporally proximate events (e.g., imaging dates, treatment timing, clinical impressions), rather than relying on manual abstractor classification (Fig. 4). Complete domain specifications are provided in Table 2 and Supplementary S1.

**Table 2.** Overview of GDM domains + data elements.

Domain	Description of elements	Temporality
Patient characteristics	Demographics, body mass index, smoking history, and performance status (Eastern Cooperative Oncology Group/Karnofsky scales); comorbidities collected as binary indicators (yes/no/unknown) mapped to ICD-10-CM codes, enabling calculation of validated indices (Charlson, NCI, Elixhauser)	For manual curation, data points are collected at diagnosis. For programmatic collection, data elements can be captured longitudinally.
SDoH	Individual-level data: insurance coverage/type, educational attainment, marital status; area-level measures linked via encrypted ZIP codes, demographics, environmental exposures, and proximity to care	Collected once to characterize patient context at diagnosis; area-level measures reflect index values at the time of data linkage.
Diagnosis	Cancer classification using ICD-O and OncoTree for histopathology and site coding; structured elements for histopathology, grade, and stage at diagnosis	Collected once per cancer diagnosis.
Imaging	Radiologic assessment information including modality (CT, MRI, PET), anatomic site, and imaging date; disease response using standard criteria (complete response, partial response, stable disease, progressive disease) with site-specific designations for mixed-response patterns	Collected for every imaging procedure/report.
Biomarkers	Clinical Laboratory Improvement Amendments–certified, clinically actionable markers; focusing on markers with established clinical utility	Collected for every biomarker test.
Surgery	Pathology report–level data for curative-intent procedures: procedure type and date, margin status, lymph node assessment, site, laterality, and histology for each specimen	Collected for every surgical procedure/pathology report.
Systemic therapy	Agent-level capture with individual tracking of each drug: start date, end date, route, and dose; enabling granular attribution of discontinuation and modifications while supporting *post hoc* regimen identification	Collected for every drug administered.
Radiotherapy	Structure for curative intent or palliative radiation: anatomic site (ICD-O topography), laterality, modality (external beam photons/protons, brachytherapy), and planning technique (3D conformal, Intensity-Modulated Radiation Therapy, stereotactic); dosimetry elements: dose per fraction (cGy), total fractions delivered, and cumulative dose	Collected for all radiation treatment episodes.
Clinical trials	Enrollment in interventional clinical trials: binary participation indicator, trial enrollment date, geographic location (country and U.S. state)	Collected for every clinical trial patient participates in.
Response/outcomes	Tumor-centric: imaging-based disease evaluation and physician-documented assessments using standard categories; person-centric: treatment completion/discontinuation with documented reasons hospitalizations, vital status, and hospice referral type	Captured longitudinally at clinically relevant time points (e.g., restaging, treatment changes, hospitalizations, death).
Derived endpoints	Calculated outcomes including overall survival, progression-free survival, disease-free survival; using disease-specific, rules-based algorithms to derive standardized, reproducible classifications	Not directly collected—calculated. Endpoints update as underlying data accrue.

### Logical model specification

**Figure 5.**
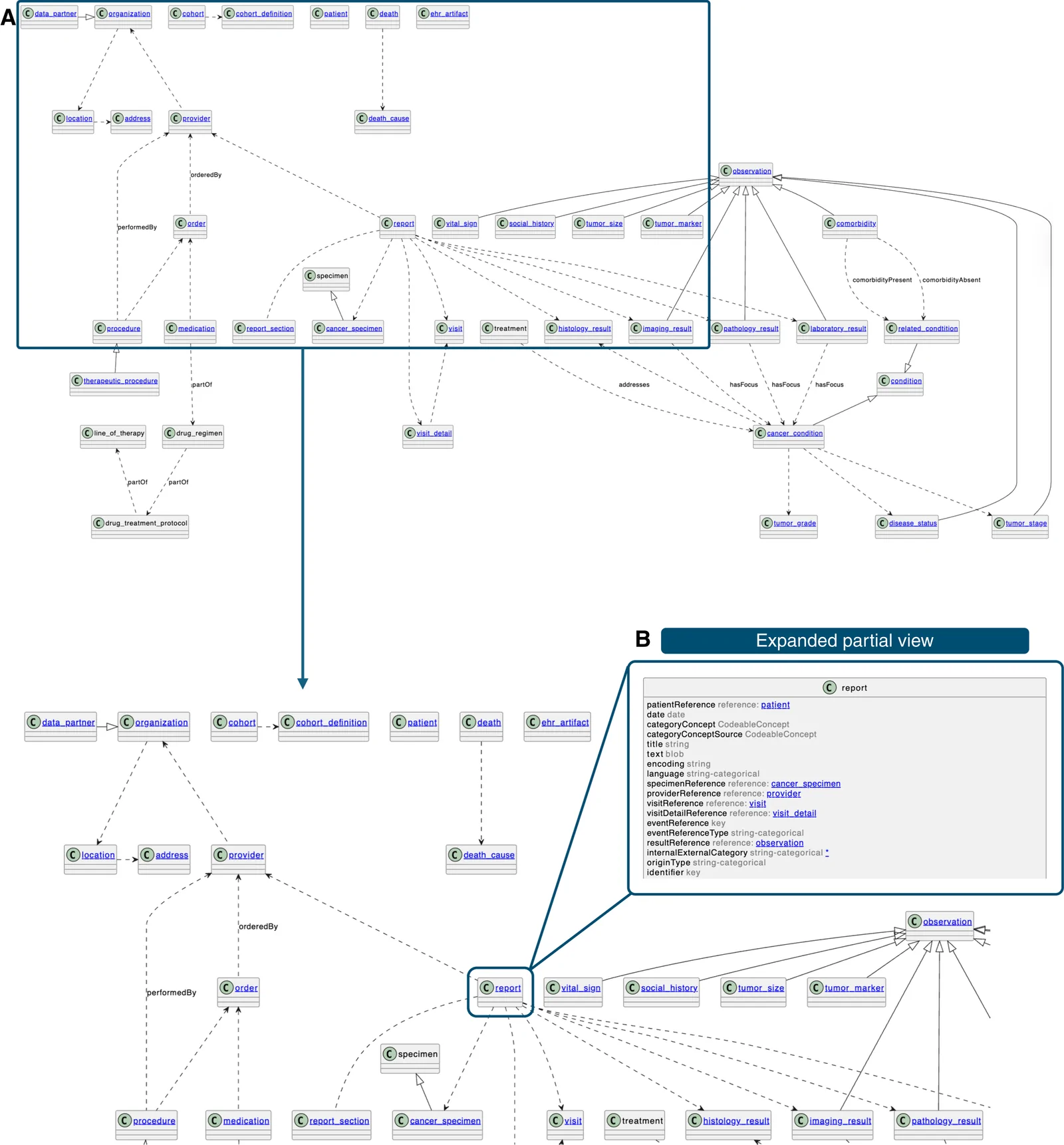
GDM logical model diagram. The GDM logical model is shown as a Unified Modeling Language diagram demonstrating relationships between different entities. **A,** Full schema view; the blue box shows foundational entities (data_partner, organization, cohort, patient, death, ehr_artifact) linked to clinical entities that represent oncology care (medication, procedure, visit, report, specimen, treatment, pathology results). Relationship cardinalities and linkages (e.g., orderedBy, performedBy) define dependencies across entities. **B,** (Blue box) Expanded view of the report entity illustrating selected attributes (e.g., patientReference, date, categoryConcept, providerReference, specimenReference, visitReference, and identifier). The model aligns with HL7 FHIR resource structures, and attributes are represented with unique model_id paths (e.g., report∼categoryConcept) to support both manual abstraction and computational derivation.

Following the specification of domain-specific variables and abstraction logic by the working groups, clinical concepts were translated into a structured data model. This process required defining not only what data to collect but also how to organize it programmatically in a manner that supports both manual abstraction and automated derivation across institutions. The GDM adopts a formal entity–attribute–data element framework in which each variable is mapped to a unique model path (model_id) representing its location in the logical model. Entities represent core oncology concepts (e.g., procedures, medications, visits, reports), whereas attributes define the data fields associated with each entity (Fig. 5). Entity–attribute pairs form the backbone of the logical model, with each path uniquely identified by a model_id using a tilde (∼) delimiter to separate entities from attributes. This convention supports attribute nesting while avoiding conflicts with dot notation used in HL7 FHIR constructs (e.g., CodeableConcept.coding.code). This logical structure supports downstream physical implementation in formats such as relational tables and enables data population from both EHR integration (computational collection) and electronic data capture tools (manual curation). These parallel pathways preserve data provenance, support auditability, and allow abstracted and derived values to coexist within the same entity. This dual-pathway design further enables the development of natural language processing pipelines to replicate human abstraction logic where feasible, facilitating scalable automation without sacrificing interpretability.

### Standards alignment, mappings, and terminology bindings

In developing the logical model specification, existing standards were systematically evaluated to ensure interoperability and alignment with established practices. HL7 FHIR served as the primary reference standard due to its alignment with existing health IT infrastructure, with additional consideration given to the OMOP CDM, the mCODE implementation guide within FHIR, and the NAACCR Data Standards and Data Dictionary. For mapping source data to harmonized representations, the GDM logical model draws inspiration from the OMOP approach and employs a framework of paired related attributes, capturing both the source concept and the corresponding standardized concept. Terminology bindings for GDM entity attributes were selected based on fitness for purpose, domain specificity, and community adoption, including SNOMED CT, LOINC, ICD-O, OncoTree, HemOncKB, NCIt, and OMOP CDM internal vocabularies. For data typing, the GDM logical model adopts HL7 FHIR data types, including the *CodeableConcept* data type for representing coded clinical concepts.

Each terminology supports a distinct function within the GDM. ICD-O supports tumor site and morphology alignment with cancer registry reporting, whereas OncoTree supports precision oncology disease groupings. SNOMED CT is an international standard used for capturing broad clinical concepts, including procedures, findings, and conditions, and LOINC is used for laboratory tests and clinical measurements. NCIt supports oncology-specific concepts, including biomarkers, therapies, disease attributes, and research-oriented cancer terminology. HemOncKB supports the structured representation of oncology drugs and regimen concepts, particularly for treatment pattern analyses. OMOP vocabularies are open-source and support observational research databases and downstream extract–transform–load (ETL) workflows. These terminologies are treated as complementary layers rather than competing vocabularies. For example, a diagnosis recorded in a local pathology system may be mapped to ICD-O for registry alignment and to OncoTree for precision oncology grouping. Similarly, a laboratory test may retain its local test name while also being mapped to LOINC. The GDM logical model structure preserves the clinical context of the original data while enabling cross-site aggregation and interoperability across FHIR, OMOP, registry, and research use cases.

### Implementation and quality assurance

The GDM is designed for flexible implementation across diverse institutional environments, supporting both prospective and retrospective data collection. Institutions may populate the model using structured EHR data, manual abstraction via electronic case report forms (eCRF), or hybrid workflows combining both approaches. The logical model structure, based on entity–attribute–data element paths, facilitates deployment as a relational physical model or as part of FHIR-compatible data services. Model deployment begins with site-specific mapping of local data systems to the GDM schema, guided by the standardized *model_id* paths and accompanying specifications. For organizations with data already mapped to OMOP, implementation should begin by identifying GDM entities and attributes that can be populated from existing OMOP tables and vocabularies, followed by a gap assessment for oncology-specific elements not consistently represented in local OMOP instances. In this setting, the GDM can serve as an oncology-specific extension to OMOP-compatible infrastructure while preserving the analytic advantages of standardized observational data models. GDM implementation can be tailored to the research question, ranging from selected domains for biomarker outcomes, treatment patterns, or response studies to full longitudinal implementation across diagnosis, treatment, response, outcomes, and person-level domains. Site implementation effort will depend on the domains included, the availability of structured data, and the use of manual, automated, or hybrid workflows. Focused GDM implementations will require clinical, data management, informatics, and analytic expertise; complete GDM deployments may also require curator capacity, terminology mapping support, Research Electronic Data Capture (REDCap) administrator support for project instantiation or site-specific modifications, database integration support, and centralized quality assurance (QA) oversight.

To ensure data quality and model fidelity, the GDM specification includes a multistaged QA framework spanning preproduction, production, and postproduction stages. In the preproduction stage, site-submitted data are validated against schema constraints, permissible values, and expected data types defined by the GDM specification. During production, data collection is governed by structured edit checks implemented directly within the eCRF, including field-level validations (e.g., permissible value ranges, required fields), calculated field logic, and branching rules to minimize entry errors and maintain internal consistency. Postproduction QA employs automated logic checks to assess cross-domain consistency and identify implausible or incomplete entries, such as ensuring treatment initiation occurs after diagnosis, confirming dose–fraction concordance in radiotherapy, and verifying the logical flow of events across forms. Following data submission from all participating sites, a centralized QA process profiles data element distributions, conducts range and plausibility checks, and audits missingness across sites to detect systematic issues. Accuracy assessment may also include source-to-standard terminology review, targeted source data verification for high-value variables, intercurator reliability checks for manually abstracted elements, and comparison of computationally derived variables against curated reference sets. Structured feedback reports are then returned to participating sites to support iterative data improvement and issue resolution.

## Results

### Clinical domain specification results

The GDM organizes data elements across clinical domains spanning initial diagnosis through treatment and long-term outcomes (Fig. 1; Table 2). Three design goals guided model development: (i) a cohort-agnostic architecture enabling consistent data capture across tumor types, (ii) integration of prospective data collection with retrospective legacy data, and (iii) extensibility to support future expansion. The patient characteristics domain captures demographics, body mass index, smoking status, and performance status (Eastern Cooperative Oncology Group/Karnofsky; refs. 21, 22). Comorbid conditions are encoded using ICD-10-CM and support the derivation of established indices (modified Elixhauser Comorbidity Index/NCI Comorbidity Index; refs. 23–26). SDoH include person-level variables (e.g., insurance type, educational attainment) and area-level metrics linked via encrypted ZIP codes, such as neighborhood socioeconomic status (Yost index; ref. 27). The incorporation of SDoH elements supports a multilevel evaluation of disparities in access to care and the social and environmental drivers of cancer outcomes while balancing data completeness, privacy, and analytic flexibility (28–32). The diagnosis domain incorporates ICD-O and OncoTree classifications to support alignment with hospital-based tumor registries and precision oncology frameworks. Staging follows the American Joint Committee on Cancer, with structured capture of histopathology, grade, and metastatic sites at diagnosis. A diagnosis date hierarchy favoring pathologic confirmation supports consistent temporal anchoring. Within the GDM, biomarker data are limited to clinically actionable nonsequencing biomarker results, including IHC, *in situ* hybridization, and other ancillary diagnostic assays, whereas somatic genomic alterations are represented through the existing GENIE genomic data schema. Cancer-specific biomarkers for 12 priority tumor types were specified based on NCCN guidelines, College of American Pathologists protocols, and Food and Drug Administration–approved companion diagnostics. These tumor types were prioritized because they represent both the largest sample volumes in the GENIE registry and the malignancies most frequently requested for GENIE-based data analyses. Additional tumor type–specific biomarkers are planned for integration in GDM v2 to expand biomarker coverage beyond the initial 12 priority malignancies. Treatment domains capture surgery at the specimen level, including margin and lymph node status, systemic therapy, with each agent tracked individually for start date, end date, route, and dose, and radiotherapy, including modality, anatomic site, dose per fraction, and cumulative dose. The response and outcomes domain captures imaging- and physician-assessed responses, treatment modifications with documented reasons, hospitalizations with reasons as a proxy for serious adverse events (SAE), and vital status determined through hierarchical linkage to EHR, tumor registry, and validated death sources, such as the National Death Index (33).

### Logical model architecture

The GDM logical model comprises 37 core entities and 753 attributes (Fig. 5). Entities, including *medication*, *visit*, *procedure*, *laboratory_result*, and *report*, were designed to reflect core aspects of oncology care and align with HL7 FHIR structures. Each data element corresponds to a precisely defined variable mapped to a unique *model_id* path. For example, *medicationdaysOfSupply* captures the days of supply for a dispensed medication, whereas *providerspecialtyConcept* represents the primary provider specialty. The dual-pathway architecture for abstraction and derivation is illustrated by the report entity. The path *reportabstractionradiologistAssessmentChangeCancerStatus* captures the radiologist’s assessment of cancer status change when extracted by human curators, whereas *reportderivationradiologistAssessmentChangeCancerStatus* captures the same information when derived computationally. The placement of both attributes under the *report* entity preserves data provenance and indicates the source from which values are obtained. This design accommodates institutional variability in data availability; for example, a manually abstracted last contact date can coexist alongside a derived last visit date computed from structured visit records, enabling flexible hybrid curation workflows. The paired-attribute framework for source-to-standard mapping is implemented across GDM entities. For example, laboratory results include both a source concept attribute (*laboratory_resultconceptSource*) and a standardized concept attribute (*laboratory_resultconcept*), both represented using the FHIR *CodeableConcept* data type. Additional details on the logical model architecture are provided in Supplementary S2.

### Treatment patterns and regimens

mCODE provides a standardized foundation for oncology data exchange but offers limited native support for longitudinal treatment pattern analysis (5). It does not explicitly model multiagent regimens, lines of therapy, or structured reasons for treatment discontinuation, constraining its utility for retrospective analyses of evolving treatment courses (5). The OMOP CDM introduces constructs such as treatment episodes and supports regimen-level representation through extensions and downstream analytics; however, modeling the complexity of precision oncology, in which therapies frequently vary in composition, overlap, or transition over time, typically requires *post hoc* inference rather than direct representation within the core model (34). In contrast, the GDM emphasizes agent-level capture of systemic therapies, including discrete start and end dates, routes of administration, and dosing information. By preserving this granular temporal structure, the GDM pairs agent-level therapy capture with standardized, model-defined derivation rules for regimens and lines of therapy, which are intended to reduce reliance on external analytic retooling or site-specific reconstruction logic.

### Outcomes ascertainment

Although OMOP has established an Oncology Module to support treatment episodes and mCODE provides structures for disease status, standardizing longitudinal response assessment remains challenging (35). These frameworks primarily capture discrete observations, often requiring downstream analytics to reconstruct disease trajectories or infer response from proxy events (34). The GDM addresses this by integrating distinct tumor-centric assessments (e.g., imaging response, physician documentation) with person-centric measures (e.g., hospitalizations, treatment discontinuations). Importantly, the GDM enables the calculation of derived endpoints through rules-based algorithms that synthesize these temporally proximate events, reducing reliance on manual classification and enabling more consistent real-world evidence generation (Fig. 4).

### Interoperability and provenance

OMOP and mCODE serve complementary but distinct purposes: OMOP CDM transforms heterogeneous data sources into a unified analytic database through extensive ETL processes, whereas mCODE defines FHIR-based profiles for clinical data exchange. The GDM addresses a different use case through its dual-pathway architecture, which distinguishes manually abstracted from computationally collected elements and explicitly preserves the provenance and logic of derived endpoints. By mapping its 37 core entities and 753 attributes to standard vocabularies (NAACCR, ICD-O, ICD-10-CM, LOINC, NCIt) and aligning with both FHIR and OMOP structures, the GDM functions as a research-optimized data model that captures both source data and the analytic transformations applied to it. Alignment with FHIR is particularly strategic given the accelerating adoption of FHIR-based health data exchange infrastructure, positioning the GDM to leverage emerging API-based data access frameworks for increasingly automated data capture and reduced reliance on manual abstraction. Standardized FHIR mappings further support integration with federated query networks and distributed analysis platforms, enabling privacy-preserving collaborative research at scale. As these technical and regulatory ecosystems mature, the GDM’s interoperability foundations create pathways toward real-time research data capture, dynamic cohort identification, and, ultimately, learning health system applications in precision oncology.

### Intended/potential applications

The GDM is designed to support diverse applications across the precision oncology research ecosystem. Within AACR Project GENIE, the model enables the harmonized collection of treatment patterns and clinical outcomes across consortium sites, facilitating comparative effectiveness research, biomarker–outcome association studies, and real-world evidence generation suitable for regulatory-grade analyses. The model’s cohort-agnostic architecture supports analyses across solid tumor diagnoses while preserving sufficient granularity for tumor-specific investigations, enabling research across both common cancers and rare malignancies.

Beyond the GENIE consortium, the adoption of the GDM provides a generalizable framework for multi-institutional precision oncology data sharing initiatives. Academic medical centers conducting investigator-initiated studies may adopt the model to standardize data collection across collaborative networks, reducing the heterogeneity that typically complicates pooled analyses. The dual-pathway architecture (manual and computational) accommodates institutions at varying stages of EHR maturity. Sites with robust structured data can emphasize automated derivation, whereas those with limited interoperability infrastructure can rely on manual abstraction workflows. Cancer registries may leverage GDM specifications to augment existing data collection with research-grade elements, particularly in domains in which current registry standards offer limited granularity, such as multiagent systemic therapy regimens and longitudinal response assessment (Fig. 3). Because the model can be implemented comprehensively or by selected domains, organizations may align adoption with specific research priorities, available source data, and local informatics capacity.

The standardization afforded by the GDM also positions it as a foundational infrastructure for federated data analysis approaches. By establishing a common data model across institutions, the framework facilitates distributed query systems and federated learning methodologies in which data remain within institutional firewalls while enabling collaborative research. This architecture addresses increasing privacy, security, and governance constraints that limit centralized data aggregation. Federated approaches leveraging the GDM may enable real-time cohort discovery, distributed validation of predictive models, and privacy-preserving multisite analyses without requiring physical data transfer, thereby expanding the scale and scope of precision oncology research while maintaining compliance with evolving data protection regulations, especially within the international context of the AACR GENIE project.

To encourage broader adoption, the complete GDM specification, including entity definitions, attribute specifications, permissible value sets, and terminology bindings, is publicly available on GitHub at https://github.com/AACR-project-GENIE. Supporting materials include data curation directives, a REDCap data dictionary, variable synopsis, and documentation to support the generation of derived variables (https://projectredcap.org/about/). Explicit mapping to established standards, where appropriate, facilitates integration with existing clinical and research systems, reducing implementation barriers for organizations already invested in these frameworks. The model’s modular design further enables selective implementation, allowing organizations to adopt specific domains relevant to their research priorities rather than requiring full-scale deployment. As the model evolves through expanding use cases, versioned releases will maintain backward compatibility to preserve existing implementations while incorporating enhancements that reflect emerging clinical practices and research needs.

## Discussion

The GDM advances oncology data standardization by addressing the fundamental tension between clinical interoperability and research granularity identified in existing frameworks. These frameworks have failed to adequately address the longitudinal complexity of precision oncology workflows. The dual pathway of the GDM architecture, distinguishing manually abstracted from computationally collected elements, balances research granularity with implementation feasibility by preserving data provenance while also accommodating institutional variability in EHR maturity and data availability. The GDM bridges this gap through strategies such as agent-level capture of systemic therapies with discrete temporal anchoring, enabling the derivation of treatment regimens and lines of therapy through standardized, model-defined rules rather than relying on *post hoc* inference or site-specific reconstruction logic. Similarly, by integrating tumor-centric assessments with person-centric outcome measures, the model enables the calculation of derived endpoints using temporally synchronized events, reducing dependence on manual classification and supporting more consistent real-world evidence generation across multiple institutions. Lastly, the explicit preservation of data provenance through parallel abstraction and derivation pathways addresses a persistent challenge in multi-institutional research: understanding how data elements were obtained and ensuring the reproducibility of derived variables.

Practical implementation considerations influenced model design at multiple levels. The cohort-agnostic architecture enables consistent data capture across tumor types, reducing implementation burden relative to disease-specific schemas while preserving extensibility for cancer-specific biomarkers and staging criteria. The modular structure allows for the selective adoption of domains aligned with specific research questions, rather than requiring comprehensive deployment across all clinical areas. By maximizing reliance on externally defined terminology standards, rather than creating proprietary concepts, the GDM facilitates integration with institutional systems already invested in these vocabularies and reduces the long-term maintenance burden associated with novel terminology development. Because these external standards follow established update cycles, they also provide a predictable mechanism for incorporating evolving clinical nomenclature, supporting ongoing model evolution as clinical practice and therapeutic landscapes change.

Several limitations should be considered when interpreting and implementing the GDM. First, the current specification focuses on solid tumors. Hematologic malignancies, which involve distinct staging systems, treatment paradigms, and response criteria, are not yet addressed within the GDM, limiting applicability across the full spectrum of malignancies. However, expansion to hematologic cancers is planned for GDM v2, with release anticipated in 2027, and will incorporate hematology-specific requirements while preserving compatibility with the existing solid tumor framework. Second, the breadth of the model can introduce an implementation burden. With more than 550 data elements, full manual deployment requires considerable abstraction effort, which may present barriers for organizations with limited data infrastructure or staffing. Although the modular design permits selective implementation, optimal prioritization of domains for specific use cases remains to be established. Third, data quality ultimately depends on clinical documentation practices, which remain heterogeneous and are evolving rapidly, especially with the adoption of ambient AI documentation tools (36). Standardization of variables and value sets may not fully resolve underlying variability in source documentation across institutions. Fourth, the current model emphasizes clinician-documented and EHR-derived data, with limited incorporation of patient-reported outcomes, quality of life measures, and functional status assessments, which remain important areas for future expansion. Fifth, SDoH are captured using a limited set of person-level variables, with area-level measures linked primarily via ZIP code for feasibility. However, ZIP code–based linkage is an imperfect proxy relative to census tract (greater contextual granularity) or county (administrative relevance) and, in a global model, is inherently U.S.-centric in the current design. Despite alignment with multiple standard vocabularies, terminology gaps also persist. No single terminology provides comprehensive coverage across all oncology domains, and the rapid emergence of novel therapeutics may outpace existing code sets, requiring ongoing terminology maintenance. Finally, although the GDM has been translated into operational specifications and implementation artifacts, systematic evaluation across participating sites remains ongoing. Future work will quantify domain-level data availability, abstraction burden, mapping completeness, QA performance, and cross-site reproducibility.

Despite these limitations, the GDM provides an open-source foundation for iterative refinement as precision oncology practice and data infrastructure mature. Planned expansion to hematologic malignancies will extend coverage to blood cancers and their distinct treatment paradigms. As oncology-specific EHR systems achieve broader adoption and provide richer structured data, the balance between abstraction and derivation pathways is expected to evolve, with corresponding updates to computational derivation rules. Ongoing GDM maintenance, versioned releases, and community access will be managed through a public GitHub repository to ensure transparency and backward compatibility as the model evolves. By providing an open-source, standards-aligned framework with explicit provenance tracking and flexible implementation pathways, the GDM establishes a foundation for scalable, reproducible precision oncology research across diverse institutional contexts. Future work will evaluate the extent to which GENIE participating sites have coverage of the critical variables defined by the GDM.

## Supplementary Material

Supplement 1 Detailed information on clinical domains

Supplement 2 Detailed information on GDM Logical Model

## Data Availability

The GDM specification, including the complete data dictionary, REDCap project files, and curated variable definitions, is available on GitHub at https://github.com/AACR-project-GENIE. The repository also includes documentation on terminology mappings (e.g., ICD-O, SNOMED CT, LOINC), permissible value sets, and logic used to generate calculated variables. Ongoing development, issue tracking, and version history will be maintained within the same repository. All other data are available from the corresponding author upon reasonable request.
