# Supplementary material for: The GENIE Data Model: A Solid Tumor, Person-Centric Framework for Scalable, Harmonized Precision Oncology Data Collection
Source: Cancer Res Commun. 2026 Sep 23;6(9):2242–55. doi: 10.1158/2767-9764.CRC-26-0148 (PMC13598700; doi:10.1158/2767-9764.CRC-26-0148)
Supplement: Supplementary file 1 [file crc-26-0148_supplement_1_suppsm1.docx]

## Supplement 1. Detailed Information on Clinical Domains

The *patient characteristics* domain captures essential clinical and demographic elements that inform prognosis and treatment eligibility. Structured fields include body mass index (BMI), smoking history, and performance status, assessed using Eastern Cooperative Oncology Group (ECOG) or Karnofsky Performance Status (KPS) scales. Comorbidities are collected using binary indicators (Yes/No/Unknown) mapped to International Classification of Diseases, Tenth Revision, Clinical Modification (ICD-10-CM) codes, enabling calculation of validated indices such as Charlson Comorbidity Index (CCI), the National Cancer Institute (NCI) Comorbidity Index, and the Elixhauser Comorbidity Index.

The *social determinants* domain captures both individual-level data (e.g., insurance coverage and type, educational attainment, marital status) and area-level measures linked to census tracts via encrypted ZIP codes. Area-level measures attainable through ZIP code linkage include neighborhood socioeconomic status (e.g., YOST Index of Socioeconomic Status), demographic composition, environmental exposures (e.g., air pollution, green space), and proximity to care (e.g., treatment site density, care deserts). Collectively, these elements enable multilevel evaluation of disparities in access and outcomes in order to contextualize variation in care delivery beyond clinical factors alone.

The *diagnosis* domain supports structured cancer classification using both the International Classification of Diseases for Oncology, 3rd Edition (ICD-O) and OncoTree, enabling alignment with tumor registry standards while enhancing granularity for genomic and precision oncology applications. ICD-O provides the foundation for histopathology and primary site coding, while OncoTree offers refined tumor type categorizations that reflect molecular and therapeutic distinctions. Additional structured elements capture histopathology, grade, and stage at diagnosis to support downstream analytic use. A diagnosis date hierarchy prioritizes pathology reports as the gold standard, followed by tumor registry data, and then radiology or laboratory findings, with derived logic specified for handling ambiguous or missing cases.

The *imaging* domain captures structured information on radiologic assessments, including modality (e.g., computed tomography (CT), magnetic resonance imaging (MRI), positron emission tomography (PET), anatomic site, and imaging date to provide temporal context. Disease response is assessed using standard oncologic criteria (complete response, partial response, stable disease, and progressive disease) and allows for site-specific designations to accommodate mixed-response patterns. This structure enables longitudinal tracking of disease burden, supports calculations such as progression-free survival, and is designed to facilitate both manual abstraction and future natural language processing (NLP)–based extraction.

The *biomarker* domain captures a focused set of Clinical Laboratory Improvement Amendments (CLIA)-certified, clinically actionable markers that are routinely used to guide treatment decisions in oncology. These include programmed death-ligand 1 (PD-L1), human epidermal growth factor receptor 2 (HER2), estrogen and progesterone receptors Estrogen receptor / progesterone receptor (ER/PR), microsatellite instability (MSI), and gene expression assays such as Oncotype DX and PAM50. This approach balances comprehensiveness with feasibility by focusing on markers with established clinical utility while allowing for future expansion as new biomarkers gain regulatory approval and clinical adoption.

The *surgery domain* captures data elements at the pathology report level, anchoring operative events to tissue specimens and providing critical surgical context for genomic sequencing analyses. The focus is on curative-intent procedures, with structured abstraction of procedure type, procedure date, margin status, and lymph node assessment. Site, laterality, and histology are documented for each reported specimen. This structure enables reproducible integration of surgical, pathologic, and molecular data, supports downstream analyses such as outcomes modeling, and minimizes manual curation burden.

The *systemic therapy* domain employs drug-level capture, with each agent tracked individually for start date, end date, route, and dose. This approach enables granular attribution of treatment discontinuation and modifications while also supporting post hoc regimen identification. This structure balances the need for detailed, agent-specific data with the analytic flexibility to model higher-order regimens, treatment classes, and combination strategies, facilitating robust clinical and translational research.

The *radiation therapy* domain uses a phase-based structure to capture delivery of curative-intent or palliative radiation. Core data elements include anatomic site (using ICD-O topography), laterality, modality (e.g., external beam photons or protons, brachytherapy), and planning technique (e.g., 3D conformal, intensity-modulated radiation therapy (IMRT), stereotactic techniques). Key dosimetry elements include dose per fraction (centi-gray [cGy]), total number of fractions delivered, and cumulative dose. This structured abstraction supports integration with other clinical domains and enables detailed assessment of radiation delivery patterns and treatment outcomes.

The *clinical trials* domain captures enrollment in therapeutic, diagnostic, screening, supportive care, or prevention trials throughout the patient’s cancer journey. For each trial enrollment, structured fields include a binary indicator of participation, the trial initiation date (defined as the first day of protocol-specified intervention), and the geographic location of the trial site (country and U.S. state, when applicable). This domain enables identification of patients who received investigational therapies and supports analyses of trial accessibility and enrollment patterns.

The *response* and *outcomes* domain captures both tumor-centric and person-centric outcomes to support longitudinal evaluation of disease burden, treatment tolerability, and survival context.

Tumor-centric outcomes are assessed through both imaging-based disease evaluation and physician-documented assessments from medical oncology visit notes. Structured capture includes response status for index lesions and distant metastatic sites using standard oncologic categories (e.g., complete response, partial response, stable disease, progression). Imaging modality, date, and anatomic site are recorded to enable consistent tracking across timepoints, including mixed-response scenarios where different anatomical sites respond differently to treatment. Cancer status documented by the treating oncologist is abstracted when available, providing additional clinical context and capturing the physician’s overall impression of disease progression or response, especially in cases where imaging is unavailable or inconclusive.

Person-centric outcomes include data elements for treatment completion, early discontinuation, and modifications, with documented reasons (e.g., toxicity, progression, patient decision, death) abstracted when available. Hospitalization is used as a pragmatic proxy for clinically significant adverse events, with fields capturing admission/discharge dates, length of stay, reason for admission (e.g., treatment-related toxicity), and 30-day readmission. Vital status is determined through a multi-source hierarchical approach leveraging electronic health records, tumor registry data, and National Death Index (NDI) linkages. Hospice referral type (inpatient or outpatient) is also captured as a potential predictor of survival and a proxy for end-of-life care transition.

The *derived endpoints* domain includes calculated outcomes such as overall survival (OS), progression-free survival (PFS), and disease-free survival (DFS). Rather than requiring abstractors to manually distinguish recurrence from progression, a distinction that is inconsistently documented in clinical records, the model captures foundational elements such as imaging dates, anatomic site-specific findings, temporal proximity to treatment, and clinical interpretation. These inputs support disease-specific, rules-based algorithms to derive standardized, reproducible classifications of recurrence and progression across cancer types, enabling scalable endpoint generation for research and real-world evidence applications.
