# Supplementary material for: The GENIE Data Model: A Solid Tumor, Person-Centric Framework for Scalable, Harmonized Precision Oncology Data Collection
Source: Cancer Res Commun. 2026 Sep 23;6(9):2242–55. doi: 10.1158/2767-9764.CRC-26-0148 (PMC13598700; doi:10.1158/2767-9764.CRC-26-0148)
Supplement: Supplementary file 2 [file crc-26-0148_supplement_2_suppsm2.docx]

**Supplement 2. Detailed information on GDM Logical Model**

**Goals and Design**

To address evolving research and data integration needs, the GENIE Data Model (GDM) was developed to better organize and coordinate data collection and downstream analysis. Whereas historical GENIE data collection efforts were largely centered on individual cancers or predefined cancer cohorts, the GDM was designed with the following overarching goals: (1) a cohort-agnostic architecture; (2) the ability to integrate both legacy data and newly collected data, (3) extensibility to accommodate future data collection requirements as they arise.

**Model Features**

The GDM is built around a set of core model entities, such as *medication*, *visit*, *laboratory_result*, and *procedure*, that reflect key aspects of oncology care. These entities are conceptually aligned with resources defined in the Health Level 7 Fast Healthcare Interoperability Resources (HL7 FHIR) standard. Each entity includes a set of attributes that capture specific details about entity. For example, the *daysOfSupply* attribute within the medication entity represents the number of days of *medication* dispensed, while the *specialtyConcept* attribute within the *provider* entity captures a provider’s primary specialty.

A fully qualified path to an attribute, referred to as *model_id* (e.g., *medication~daysOfSupply*), is a central construct in the GDM. The tilde (~) character is used to separate entities from attributes and to represent nested attributes. This convention was chosen to avoid conflicts with dot-notation commonly used in HL7 FHIR constructs (e.g., *CodeableConcept.coding.code*).

Together, entities and their attributes constitute the GDM logical data model. This logical model can be implemented as a physical data model. For example, *daysOfSupply* may be implemented as a column within a relational database table representing the *medication* entity. The GDM physical model can be populated either through programmatic ingestion of electronic health record(EHR) data or through manually collected Case Report Form (CRF) data.

**Data Elements**

A third core construct of the GDM is the *data element*, which governs data collection within the model. Consistent with published definitions, a data element is defined as a precisely specified question (variable) paired with a defined set of allowable responses, intended for use across multiple datasets or studies.^37^ Data elements may correspond to historically collected variables or to planned future variables for which data have not yet been collected. Within the GDM, each data element (identified by a *data_element_id*) is mapped to a corresponding entity-attribute path (*model_id*) in the logical model.

**Abstraction and Derivation**

To coordinate CRF-driven data collection with programmatic data ingestion, the GDM explicitly distinguished between data abstraction and data derivation through dedicated attributes. D*ata abstraction* refers to human-driven data collection using CRFs, which relies on curator review and interpretation of clinical records. In contrast, *data derivation* refers to computationally generated data obtained from structured EHR fields or other source systems without manual input. For example, *last contact date* may be abstracted through curator review of all available clinical documentation, whereas *last visit date* may be derived algorithmically as the most recent recorded patient encounter in the EHR. Both abstraction- and derivation-based attributes can coexist within the same entity, enabling transparent provenance tracking and flexible hybrid workflows

**Standards Alignment and Terminology**:

In developing the GDM, multiple standards were considered. HL7 FHIR served as the primary reference, with additional considerations given to the Observational Medical Outcomes Partnership Common Data Model (OMOP CDM), minimal Common Oncology Data Elements (mCODE) implementation guide within FHIR, and the North American Association of Central Cancer Registries (NAACCR) Data Standards and Data Dictionary.

Terminology bindings for GDM entity attributes draw from established vocabularies, including Systematized Nomenclature of Medicine-Clinical Terms (SNOMED CT), Logical Observation Identifiers Names and Codes (LOINC), International Classification of Diseases for Oncology (ICD-O), Hematology Oncology Knowledge Base(HemOncKB), National Cancer Institute Thesaurus (NCIt), and OMOP internal vocabularies (e.g., for visit type classification).

For data types, the GDM adopts HL7 FHIR data types and follows the FHIR approach to representing coded concepts using the *CodeableConcept* data type. To support harmonization of source data, the GDM incorporates an OMOP-inspired paired-attribute framework, in which each concept may be represented by both a source concept attribute (e.g., *laboratory_result~conceptSource*) and a standardized concept attribute (e.g., *laboratory_result~concept*). Both attributes are represented using the *CodeableConcept* data type.

**Natural Language Processing**

The GDM was designed with the expectation that Natural Language Processing (NLP) will play a central role in abstracting information from unstructured clinical reports. The *report* entity includes numerous abstraction and derivation attributes to support this approach. For example, *report~abstraction~radiologistAssessmentChangeCancerStatus* captures a radiologist’s assessment of cancer status change when extracted by a human curator, while *report~derivation~radiologistAssessmentChangeCancerStatus* represents the same clinical concept when derived computationally. In both cases, the nested attribute (*radiologistAssessmentChangeCancerStatus*) remains consistent, with the abstraction or derivation context explicitly encoded in the model. The placement of these attributes under the *report* entity indicates the source of information used for abstraction (human) or derivation (computational). The GDM also supports structured clinical reporting through the inclusion of a *report_section* entity, enabling alignment between narrative and structure report content.
